# Tuning Up the Old Brain with New Tricks: Attention Training via Neurofeedback

**DOI:** 10.3389/fnagi.2017.00052

**Published:** 2017-03-13

**Authors:** Yang Jiang, Reza Abiri, Xiaopeng Zhao

**Affiliations:** ^1^Aging Brain and Cognition Laboratory, Department of Behavioral Science, College of Medicine, University of KentuckyLexington, KY, USA; ^2^Sanders-Brown Center on Aging, College of Medicine, University of KentuckyLexington, KY, USA; ^3^Department of Mechanical, Aerospace, and Biomedical Engineering, University of TennesseeKnoxville, TN, USA; ^4^Institute for Medical Engineering and Science, Massachusetts Institute of TechnologyCambridge, MA, USA

**Keywords:** EEG, ERP, biofeedback, brain modulation, SVM, cognitive aging, BCI

## Abstract

Neurofeedback (NF) is a form of biofeedback that uses real-time (RT) modulation of brain activity to enhance brain function and behavioral performance. Recent advances in Brain-Computer Interfaces (BCI) and cognitive training (CT) have provided new tools and evidence that NF improves cognitive functions, such as attention and working memory (WM), beyond what is provided by traditional CT. More published studies have demonstrated the efficacy of NF, particularly for treating attention deficit hyperactivity disorder (ADHD) in children. In contrast, there have been fewer studies done in older adults with or without cognitive impairment, with some notable exceptions. The focus of this review is to summarize current success in RT NF training of older brains aiming to match those of younger brains during attention/WM tasks. We also outline potential future advances in RT brainwave-based NF for improving attention training in older populations. The rapid growth in wireless recording of brain activity, machine learning classification and brain network analysis provides new tools for combating cognitive decline and brain aging in older adults. We optimistically conclude that NF, combined with new neuro-markers (event-related potentials and connectivity) and traditional features, promises to provide new hope for brain and CT in the growing older population.

The ability to focus attention, encode and maintain information are among the brain’s most important cognitive functions. Attention is a central component of cognitive ability. Measurements of neural activity have become strong predictors of cognitive impairments in persons afflicted with various kinds of cognitive deficits. Lapses in attention can impair memory and behavioral performance.

Complaints about declined attention and memory are common in healthy and cognitively intact older adults during brain aging. Deficits in attention and memory are also the most common symptoms in older adults with dementia such as Alzheimer’s disease (AD), Parkinson’s, or vascular dementia (VD). Old-age dementia affects patients’ daily lives with memory loss and cognitive impairments. The most common early symptoms of AD are problems with short-term memory (Reiman et al., 2011). Since there is no effective drug treatment thus far to stop cognitive decline, attention training has become an increasingly attractive option. The effectiveness of cognitive rehabilitation including attention training has been under debate for decades. A recent review has shown evidence that attention training enhances attention and memory with moderate success (Cicerone et al., 2011). Since attention is a core function for multitude of cognitive processes (e.g., memory and perception), most cognitive training (CT) programs seek to increase the existing attentional capacity.

## Brain-Computer Interface (BCI)

Research on Brain-Computer Interface (BCI), also known as brain-machine interface (BMI), dates back to the 1960s (Miranda et al., 2015). BCIs and BMIs are systems that utilize recorded brain activity to communicate between the brain and computers in order to control the environment in a manner that is compatible with the intentions of humans and to receive feedback from environment. In BCI, the brain activity is recorded through various neuroimaging methods, which can be categorized in two groups: invasive and noninvasive. Electrocorticography (ECoG) and Electroencephalography (EEG) are known as the most common invasive and noninvasive methods, respectively (Nicolas-Alonso and Gomez-Gil, 2012). A closed-loop BCI system with real-time (RT) modulation and communication can not only be employed in directly controlling external devices, but can also be utilized as a biofeedback platform to improve and enhance the cognitive abilities of individuals (Chaudhary et al., 2016).

## Neurofeedback (NF)

Neurofeedback (NF) is a form of EEG biofeedback used to successfully improve cognitive and physical performance of humans (Daly and Wolpaw, 2008; Pfurtscheller et al., 2008; Machado et al., 2013; Broccard et al., 2014; Chaudhary et al., 2016). Cognitive enhancement training after mild traumatic brain injury (mTBI) has been shown to increase focused attention and memory, thus improving the patient’s performance in daily life (Cicerone et al., 2011). More convincing evidence of effectiveness of working memory (WM) and executive-control training in older adults comes from a meta-analysis by Karbach and Verhaeghen (2014). They examined 61 independent samples in adults over the age of 60. Cognitive interventions resulted in significant improvement in performance on the trained task and untrained similar tasks. There was even a small but significant training-induced improvement in untrained tasks in a different domain, demonstrating that training has transferred far into learning.

The presently popular CT method is attention process training (APT; Sohlberg et al., 2000), which also includes WM components. While efficacy of these methods differs, all have been reported to enhance performance in focused attention tasks, cognitive function and WM tasks. Some attention training showed a learning transferable effect, i.e., improved performance in untrained tasks (Sinotte and Coelho, 2007; Westerberg et al., 2007; Cicerone et al., 2011; Kuo et al., 2014). However, evidence for improvement in everyday life utilizing cognition has been limited thus far, which provides impetus for developing better and time-efficient methods to directly train neural processes underlying attention. Cicerone et al. (2011) concluded that attention seems to train better than other domains of cognition. For treatment of children with attention deficit/hyperactivity disorder (ADHD), NF has been shown to be a better intervention than traditional attention (Hurt et al., 2014; Steiner et al., 2014) or WM training (YuLeung To et al., 2016). Notice though, evidence from meta-analyses of randomized controlled trials fails to support NF as an effective treatment for ADHD in children and adolescents. The significant treatment results only occur in the outcome measures that are not properly blinded (Cortese et al., 2016). In a comprehensive review on EEG-based BCI NF, Ordikhani-Seyedlar et al. (2016) pointed out that, despite amazing progress, a major challenge for attentional training via NF is improving signal processing algorithms that dissociate brainwaves of attended from those of unattended items.

## What’s Special About Cognitive and Brain Aging?

### Challenges of Training Older Adults

The challenge of attentional training in older adults is that measurement of CT is often confounded with multiple factors, such as individual differences that tend to increase with age. These factors include individual differences in brain aging associated with visual attention (Monge et al., 2016), attention capture to rewarding objects (e.g., a parieto-occipital electrophysiological responses; Donohue et al., 2016), WM and performance (Parasuraman and Jiang, 2012), learning transfer beyond trained tasks (Greenwood and Parasuraman, 2016), and placebo effects where performance of older adults is simply improved by participating in CT (Foroughi et al., 2016).

Unlike ADHD in children, prominent cognitive deficits in aging brain occur in the switching and division of attention, whereas phasic arousal and focused attention to stimulus features are only minimally affected in the early stages of AD. For instance, selective attention deficit is one of the first cognitive indicators of neocortical dysfunction in early AD (Parasuraman and Haxby, 1993). Despite many challenges, comprehensive treatment in patients with mild cognitive impairments (MCI), including NF training, diet and fitness programs, has shown great promise in cognitive improvement (Bredesen et al., 2016; Fotuhi et al., 2016). Importantly, efficacy of all NF training schemes will need to be rigorously tested by comparing independent measures and sensitive indicators of attention and WM before and after attention training.

### Training Attention and Working Memory to Prevent AD Risk

Besides attention, neural mechanisms underlying short-term memory (e.g., WM) undergo a significant early change in aging (Lawson et al., 2007) and in AD dementia patients (Grady et al., 2001). The memory decline also includes neural mechanisms underlying repetition learning, a form of implicit memory (Jiang et al., 1999, 2009). Early AD/MCI manifests itself in loss of short-term memory but retention of intact long-term memory. Since WM and attention shared common neural mechanisms (e.g., Gazzaley and Nobre, 2012), enhancement of attention improves encoding, maintenance and retrieval of items held in WM for online usage.

It is critical that future attention training via NF in older adults targets specific neuro-markers underlying attention/WM and related performance. For instance, a short-term memory paradigm based on well-established single-cell electrophysiological experiments in primates (Miller and Desimone, 1994) was developed for human neuroimaging using functional magnetic resonance imaging (fMRI; Jiang et al., 2000, 2016), and EEG (Lawson et al., 2007; Guo et al., 2008). Using the short-term memory paradigm, the same patterns of brain responses in older adults and MCI have been validated in a Chinese cohort of older adults (Yu et al., 2016). Testing a cohort of cognitively normal older adults in the U.S., Jiang et al. (2016) reported that increased bilateral parietal connectivity during the short-term memory task is correlated with higher Tau levels in Cerebrospinal fluid (CSF) biomarker, indicating increased risk of AD. CSF Tau AD biomarker did not show such a link to brain connectivity during resting state, but only when the brain was challenged with a cognitive task. In contrast to CSF AD biomarkers, which showed no associations with cognitive status in normal these adults, functional brain connectivity between left temporal and parietal gyri during the memory task strongly correlated with overall cognitive status. Furthermore, modulating cognitive neuro-markers validated by AD biomarkers (e.g., CSF) should be even more effective approach in cognitive improvement specifically targeting aging brain.

## Advancement in Brain Training Methodology

The effectiveness of CT has been subject to doubt for decades. Recently, brain training has experienced a renaissance due to new advances in brain imaging, BCI and advanced analytical tools. Applying state-of-the-art RT classification tools, a recent study used fMRI to provide NF during attention training and successfully improved visual attention and behavioral performance (deBettencourt et al., 2015). This study also aimed to increase the efficiency of attention so that a person may sustain high attention to a task for a longer period of time to improve memory, which is the key element for improvement in cognitive aging.

### EEG Based Neurofeedback Training

EEG has been in use since 1930s (Adrian and Matthews, 1934). What are the new tricks for improving brain training? For decades, scalp EEG studies of AD mainly focused on characterizing clinically-evident disease stages rather than preclinical AD. The important EEG components in human adults are the delta (<4 Hz), theta (4–7 Hz), alpha (8–13 Hz), and beta waves (>13 Hz). Theta and delta waves are known as slow waves. Alpha waves, sourced in frontal sites including anterior cingulate cortex, are related to attention, WM, and related performance in humans. It has been shown to be sensitive to suppression of unattended stimuli (Händel et al., 2011). EEG theta oscillations are also related to hippocampal activity during WM (Tesche and Karhu, 2000). Spatial attention is a constant theta-rhythmic sampling process implemented through gamma-band synchrony (Landau et al., 2015).

NF using traditional EEG and new EEG neuro-markers has demonstrated success in recent times, especially in children with ADHD in the 6–12 age range (Holtmann et al., 2014). Relative power of theta, alpha, beta, theta/alpha and theta/beta ratios were applied during successful training in children with ADHD (Hillard et al., 2013), and by using Theta/Alpha Ratio (Steiner et al., 2014). Similar success has been shown in NF training using theta/Alpha ratio in children age 6–12 with a learning disorder (Fernández et al., 2016). However, in slightly older children with ADHD, failure of improvement was reported in a double-blind placebo-controlled study (Vollebregt et al., 2014), which theta/beta ratio and theta/alpha ratio were utilized. The outcome measures of neurocognitive performance before and after treatment failed to show improvement, possibly due to sensitivity of the outcome measures and other study limitations.

As for NF training in the older brains, the seminal work by Angelakis et al. (2007) applied EEG NF in the older population and showed improved processing speed and executive functions (EFs). Additional success has been reported using EEG-based NF for attention training and WM in young adults (Egner and Gruzelier, 2001; Zoefel et al., 2011; Ros et al., 2013, 2014), in post-traumatic stress disorder (Ros et al., 2016), and in older dementia patients (e.g., Surmeli et al., 2016). We summarize some of the recent studies on attention or WM training in older and younger adults in Table 1.

**Table 1:** **Selective studies on attention or working memory training using neurofeedback (NF) in older and younger adults**.

Publications	Age group	Mean age (Range in years)	Tasks (number of participants)	Neuro-markers	Improvement?
Angelakis et al. (2007)	Older adult (H)	74 (70–78)	Memory, Processing speed; NFT and SNFT (*n* = 3 each group/condition)	Alpha Frequency	Y (Speed and EF)
Lecomte and Juhel (2011)	Older adult (H)	75.25 (65–85)	Working Memory; NFT and SNFT	Alpha, Theta	Mixed (Memory)
Becerra et al. (2012)	Older adult (H)	65.8 (60–84)	Executive Function + Memory Tasks; NFT and SNFT (*n* = 7 each)	Theta	Y (WM)
Wang and Hsieh (2013)	Young + Older (H)	21.8 (21–25); 64.6 (61–67)	Attentional Network + Recognition Task; NFT and SNFT (*n* = 8 each)	Fronto-midline Theta	Y (Attention and WM)
Staufenbiel et al. (2014)	Older adult (H)	67.8	Intelligence + Memory Task; Beta and Gamma Groups (*n* = 10 each)	Gamma, Beta	Y (WM)
Luijmes et al. (2016)	Older adult (AD)	64–78	Cognitive Examination; NFT (*n* = 10)	Delta, Theta, Alpha, Beta	Y (WM)
Reis et al. (2016)	Older adult (H)	65.97 (59.3–72.6)	Working Memory Task; NFT (*n* = 9), NFCT (*n* = 8), CT (*n* = 7), SNFT (*n* = 6)	Theta, Alpha	Y (WM)
Surmeli et al. (2016)	Older adult (AD + VD)	68.9 (58–79)	EEG-guided NFT (*n* = 20); within subjects’ design	Inhibit Theta, Alpha, Beta (21–32 Hz)	Y* (MMSE)
Egner and Gruzelier (2001)	Young adult (H)	22.1	Oddball Task; NFT (*n* = 22)	P300 ERP, beta1, SMR learning	Y (Attention)
Zoefel et al. (2011)	Young adult (H)	23 (21–26)	Mental Rotation Task; NFT (*n* = 14), SNF (*n* = 10)	Upper Alpha Frequency	Y (Mental rotation)
Ros et al. (2013)	Adult (H)	32.6 (22–42)	Attentional and Oddball Tasks; NFT and SNFT (*n* = 17 each)	fMRI, Alpha frequency	Y (Attention)
deBettencourt et al. (2015)	Adult (H)	20.3	Selective attention task (superimposed images Figure 1; *n* = 16 each condition)	Real-time fMRI	Y (Attention)

H, Healthy participants; LD, Leaning disorder; ADHD, attention deficit hyperactivity disorder; AD, Alzheimer’s disease; VD, vascular dementia; mTBI, mild traumatic brain injury; NFT, neurofeedback training group; SNFT, sham neurofeedback group; CT, cognitive training; NFCT, neurofeedback and cognitive training group; RT, real-time; EF, executive function; WM, working memory; Y*, some of the participants; MMSE, Mini Mental State Exams.

## Novel Neuro-Markers for Cognitive Change in Old Age

Recent work has identified neurosynaptic changes as one of the earliest biomarkers of preclinical AD, appearing before onset of tau-mediated neuronal injury or brain structure changes (Jack et al., 2011; Sperling et al., 2011). EEG recordings directly measure post-synaptic potentials and are able to detect these early changes. It has been shown that measured synchronized electrophysiological signals during sleep and rest can be used as neurophysiological biomarkers for the early detection and classification of dementias (Al-Qazzaz et al., 2014).

### Cognitive ERP Markers

The averaged EEG signals, i.e., ERPs during cognitive events known as cognitive ERP, provides a promising neuro-markers for indexing changes of neural mechanisms underlying cognition and memory (Olichney et al., 2011). Altered amplitude or latency of ERP signals in patients with AD have been reported (Jackson and Snyder, 2008). In addition, abnormal cognitive ERP P600 during a word memory task in a small sample of older adults with preclinical AD has been reported (Olichney et al., 2008, 2011, 2013). Similar to EEG, cognitive ERP biomarkers are a noninvasive and more cost-effective method than CSF, PET biomarkers for early diagnosis of AD. Cognitive ERP biomarkers are sensitive to WM and attention deficits before conventional biomarkers of AD can be detected by behavioral performance changes (Li et al., 2017).

### Brain Network-Based Neuromarkers

Recent evidence in animal models and neuroimaging also points to brain connectivity networks as novel neuro-markers for indexing early deficits in AD risk. Aß peptides disrupt neural activity at the synaptic level and induce aberrant activity patterns in neural network circuits within and between brain regions in animal models (Palop and Mucke, 2010). Resting-state network based technology using EEG has demonstrated abnormalities in cognitive ability (Babiloni et al., 2006a,b, 2009, 2010; Prichep et al., 2006; Prichep, 2007). Patterns of functional brain connectivity in humans are highly predictive of cognitive performance (Hachinski et al., 2006; Finn et al., 2015). Recent fMRI work shows that brain connectivity correlates differentially with CSF AD biomarkers both during resting state and cognitive tasks (Jiang et al., 2016).

## Advanced Real-Time EEG Analysis

### Advanced Network Causality Analysis in Old Brains

**Figure 1:**
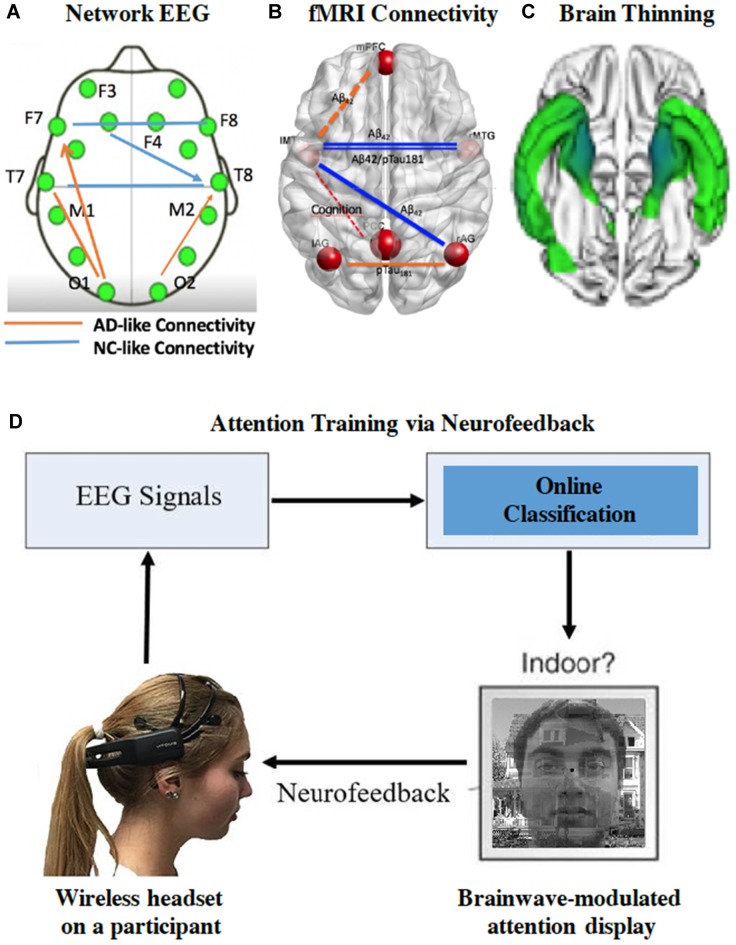
**The individual brainwaves are neuromarkers for cognitive states. (A)** The network electroencephalography (EEG) causality analysis of brain connectivity differentiates healthy older adults (NC) from early Alzheimer’s disease (AD) patients (Adaptation of McBride et al., 2015). **(B)** Functional magnetic resonance imaging (fMRI) brain network analysis from cognitive normal participants in the University of Kentucky cohort (bilateral anterior temporal connectivity correlates with early AD risk; Jiang et al., 2016). **(C)** Cortical thinning in temporal cortices (*n* = 24) was seen in older patients with very early stage of AD at the Unviersity of Kentucky. **(D)** The integrated platform for EEG/ERP closed-looped neurofeedback (NF) during attention training. Facial images are used with permission.

As brain ages, Aβ plaques form within distinct regions of the brain’s default-mode network (Buckner and Vincent, 2007). Other factors such as age, genes and cognitive reserve in older individuals also add to the complexity of predicting AD risk in an individual. While fMRI connectivity is a good indicator for network of brain circuits, EEG offers superior temporal resolution, simpler and more affordable application in clinical settings. For instance, the network EEG neuro-marker can be a predictive neuro-marker for AD risk (Stam, 2014). Growing evidence has shown that brain functional connectivity changes in dementia can be identified in EEG recordings (McBride et al., 2013, 2014, 2015; Sargolzaei et al., 2015). Engels et al. (2015) reported decreasing functional connectivity in the posterior regions, together with a shifted hub location from posterior to central regions with increasing AD severity. In addition, causality analysis is taking the center stage. For instance, causality analysis based on the Granger method was used to infer synaptic transmission, which was reflected in EEG measurement and information flow in the neural network (Trongnetrpunya et al., 2016). The Granger causality algorithm was also used to assess brain connectivity in scalp EEG with success (e.g., Barrett et al., 2012). They identified significant increases in bidirectional Granger causality during loss-of-consciousness, especially in the beta and gamma frequency ranges. In contrast to Granger causality analysis (Bressler and Seth, 2011), Sugihara et al. (2012) proposed the dynamic causation concept (Deyle and Sugihara, 2011). A novel brain functional connectivity marker based on Sugihara’s causality definition (McBride et al., 2015) has been developed to allow characterization of brain network changes beyond traditional features at localized brain sites. Figure 1 illustrates the consistent connectivity changes in older brain using measures of network EEG (Figure 1A), fMRI connectivity (Figure 1B), and white matter integrity (Figure 1C) in the aging cohort followed by Sanders-Brown Center on Aging at Bluegrass Region in Central Kentucky. These findings open up new ways in training older brainwaves during tasks toward those seen in younger brains.

### Real-Time EEG and fMRI Based NF Training

Brain training using frequency based EEG features (alpha, theta, or theta/beta power) is commonly used in the NF attentional training. Applying EEG-based NF for improving cognitive performance has been reviewed comprehensively by Gruzelier (2014). New studies using EEG neuro-markers beyond frequency neuromarkers have been showing new promise. The brain dynamics (EEG long-range temporal correlations) can be modulated with stimulation in an involuntary manner, which is an excitation-inhibition balance change achieved by the closed-loop neuro-regulation (Ros et al., 2014; Reis et al., 2016; Zhigalov et al., 2016). Using simultaneous EEG and fMRI, Zotev et al. (2014) demonstrated potential applications of novel NF paradigms for treating mental disorders including cognitive aging. Liu et al. (2015) proposed a fractal dimension (FD)-based NF training protocol with adaptive algorithm. The FD-based NF does not require before-training recording. The efficiency of the FD-based NF training in comparison with traditional individual theta/beta based NF training is assessed for focused attention and test of attentional vigilance. They reported that after NF training participants from FD-based training group have similar or better test performance than the one from the ratio-based group.

RT classification of complex brain activity has been an exciting development. A recent study demonstrated that NF using “RT” fMRI during attention training can be used to successfully improve visual attention (deBettencourt et al., 2015). Although fMRI-based NF has definite advantage of revealing where the modulation occurs in the brain, the use of MRI requires participants to remain motionless during training sessions. Additionally, fMRI technique indirectly measures neural activity by quantifying blood oxygen levels, and is costly as well. Thus, there is renewed interest in developing user-friendly advanced EEG-based NF. Aided by new EEG recording technologies such as wireless EEG headsets (e.g., Emotiv device featured in Figure 1D) and gaming devices, more investigations on NF via brain network that utilize faster RT classification analysis have emerged. The following example is how the combination of EEG frequency and advanced EEG features analysis (e.g., spectral entropy) are used to modulate brain activity for better attention training.

### Feature Extraction and Close-Loop BCI Neurofeedback

The employed features from collected EEG data for focused target in initial testing and validation of the platform are oscillation activity of delta, theta, alpha, beta and gamma bands, as well as the spectral entropy. Additionally, spectral entropy is information entropy that is able to quantify the spectral complexity of an uncertain system. Modeled after successful fMRI paradigm of attention training (deBettencourt et al., 2015), a new noninvasive BCI system has been developed using scalp EEG to decode the sustained attention level of a human participant with an efficient NF based on his/her level of attention-related brain signals. Figure 1D shows a schematic of the attention-based NF system. During the test, scalp EEG signals of a subject are recorded via wireless EEG headset while the subject is focusing on a sequence of superimposed images. Each image is a mixture of two transparent pictures from two categories (Scene vs. Face). At a given time, an observer is instructed to pay attention to the task-relevant stimulus (e.g., scene) and ignore the irrelevant stimulus (e.g., face). The level of the attentional state of the subject towards the targeted task-relevant stimulus will be determined from a regression model of the EEG signals in RT. If the attentional level is high for the current image, then the subject is rewarded with improved sharpness of the target stimulus in the next composite image. Thus, the dynamics of changing superimposed images serves as rewarding positive NF, encouraging the subject to focus his/her attention on the target visual stimuli. Several EEG and EPR-based BCI platforms for prosthetic control (Abiri et al., 2015a, 2016a) and NF of attention training have been developed in adults (Abiri et al., 2015b, 2016b), which is promising as foundation for the next step of testing in older adults using well designed and controlled experiments.

## Conclusion

This review has summarized the rapid growth in BCI technology, online machine learning classification, and advanced brain network analysis, which are some of the exciting new methods in combatting cognitive and brain training in older adults. BCI-based NF training provides new methods for instant reward of brainwave patterns associated with better cognitive functions or younger brains. We envision great progress will occur in brain training of attention and short-term memory, core cognitive abilities, by modulating non-invasively recorded electrical brain activity via RT NF in older adults. This is an exciting time for developing CT in older adults. With the work reviewed here, we conclude that RT NF, combining traditional frequency and new neuro-markers, promises to provide new hope for brain and CT in older adults.

## Author Contributions

YJ wrote the first draft. RA and XZ made significant and original contribution.

## Funding

Part of the work was supported by National Institute of Aging, Henry Jackson Foundation and NeuroNET.

## Conflict of Interest Statement

The authors declare that the research was conducted in the absence of any commercial or financial relationships that could be construed as a potential conflict of interest.
